# An Unbiased Systems Genetics Approach to Mapping Genetic Loci Modulating Susceptibility to Severe Streptococcal Sepsis

**DOI:** 10.1371/journal.ppat.1000042

**Published:** 2008-04-18

**Authors:** Nourtan F. Abdeltawab, Ramy K. Aziz, Rita Kansal, Sarah L. Rowe, Yin Su, Lidia Gardner, Charity Brannen, Mohammed M. Nooh, Ramy R. Attia, Hossam A. Abdelsamed, William L. Taylor, Lu Lu, Robert W. Williams, Malak Kotb

**Affiliations:** 1 Mid-South Center for Biodefense and Security, The University of Tennessee Health Science Center, Memphis, Tennessee, United States of America; 2 Department of Ophthalmology, The University of Tennessee Health Science Center, Memphis, Tennessee, United States of America; 3 VA Medical Center, Memphis, Tennessee, United States of America; 4 College of Pharmacy, Cairo University, Giza, Egypt; 5 Department of Molecular Sciences, The University of Tennessee Health Science Center, Memphis, Tennessee, United States of America; 6 Molecular Resource Center, The University of Tennessee Health Science Center, Memphis, Tennessee, United States of America; 7 Department of Anatomy and Neurobiology, The University of Tennessee Health Science Center, Memphis, Tennessee, United States of America; Massachusetts General Hospital, United States of America

## Abstract

Striking individual differences in severity of group A streptococcal (GAS) sepsis have been noted, even among patients infected with the same bacterial strain. We had provided evidence that HLA class II allelic variation contributes significantly to differences in systemic disease severity by modulating host responses to streptococcal superantigens. Inasmuch as the bacteria produce additional virulence factors that participate in the pathogenesis of this complex disease, we sought to identify additional gene networks modulating GAS sepsis. Accordingly, we applied a systems genetics approach using a panel of advanced recombinant inbred mice. By analyzing disease phenotypes in the context of mice genotypes we identified a highly significant quantitative trait locus (QTL) on Chromosome 2 between 22 and 34 Mb that strongly predicts disease severity, accounting for 25%–30% of variance. This QTL harbors several polymorphic genes known to regulate immune responses to bacterial infections. We evaluated candidate genes within this QTL using multiple parameters that included linkage, gene ontology, variation in gene expression, cocitation networks, and biological relevance, and identified interleukin1 alpha and prostaglandin E synthases pathways as key networks involved in modulating GAS sepsis severity. The association of GAS sepsis with multiple pathways underscores the complexity of traits modulating GAS sepsis and provides a powerful approach for analyzing interactive traits affecting outcomes of other infectious diseases.

## Author Summary

Group A streptococci (GAS) cause a wide variety of human diseases ranging from mild pharyngitis to streptococcal toxic shock syndrome and necrotizing faciitis. Our previous studies have shown that host immunogenetic variation can dictate the clinical outcome of GAS sepsis. As in most human disease, GAS sepsis is likely to be affected by complex interactions between more than one polymorphic gene. We addressed this issue in our study where we present an approach that allowed us to identify multi genetic factors that likely contribute to sepsis severity. We mapped susceptibility to severe GAS sepsis to quantitative trait loci on Chromosome 2 using a panel of genetically diverse inbred mice. The mapped regions have high single nucleotide polymorphism (SNP) density that harbor genes known to play an important role in innate immune response to bacteria. Several of those genes are differentially expressed between susceptible and resistant strains of mice. Our overall approach of systematic dissection of genetic and molecular basis of host susceptibility is not unique to GAS infections, but can be applied to other infectious diseases to develop better diagnostics, design effective therapeutics and predict disease severity based on a set of genetic and soluble biomarkers.

## Introduction

Infectious diseases, like most human diseases, are modulated by complex traits. Susceptibility and clinical outcomes of infections are often a manifestation of interactions between the host's complex traits and the pathogen's virulence components. Identification of genes and molecular networks that influence host responses to infectious agents can provide a disease road map that would focus the discovery of effective diagnostics and therapeutics.

Group A streptococci (GAS) are classified on the basis of surface M protein antigens into more than 100 serotypes, but recent studies showed a high degree of diversification within a serotype that is driven primarily by horizontal gene transfer [1]–[3]. It is widely believed that such events are responsible for the emergence of highly virulent strains in the 1980's, including a hypervirulent M1T1 clonal strain, coinciding with the resurgence of severe invasive GAS infections associated with streptococcal toxic shock syndrome (STSS) and necrotizing fasciitis (NF), also known as the “flesh eating” disease [4]. We showed that this hypervirulent M1T1 clinical strain can cause sore throat or mild bacteremia and erysipelas in some patients, while in others it can cause STSS and NF [5]. These findings suggested a strong role for host factors in modulating the outcome of infection by this highly virulent strain. Indeed, we found that allelic variations in host HLA class II haplotypes are associated with markedly different outcomes of GAS sepsis in humans [6],[7]. We confirmed these associations using HLA transgenic mice that were expressing different HLA class II alleles [8]. Inasmuch as STSS pathogenesis is largely mediated by streptococcal superantigens (Strep SAgs), and because HLA class II molecules serve as SAg receptors, such an association was expected. However, GAS is rich in many other virulence factors, and we believe that some of those virulence factors, beside SAgs, may also interact with additional host factors to modulate host responses to GAS infections.

To identify additional host factors involved in GAS severity we needed an approach that allowed us to uncover the effect of interactions between complex, polymorphic genetic traits that may modulate sepsis outcomes. Previous animal models for GAS sepsis included various regular conventional inbred mouse strains [9],[10], congenic strains [11], and HLA transgenic mice [8],[12]. These models confirmed that host genetic variability can have a strong effect on infection outcome. Despite their significant utility, these mouse models do not offer the genetic diversity that is characteristic of the human population.

In this study, we used a panel of advanced recombinant inbred (ARI) mice as a genetically diverse, segregating reference population that affords a powerful tool for systems genetics approaches. Recombinant inbred (RI) strains have been successfully used to map quantitative trait loci (QTLs) associated with various phenotypes and diseases [13]–[20]. We used the BXD panel of ARI mice derived from C57BL/6J (B6) and DBA/2J (D2) strains and consisting of homozygous, inbred BXD lines, each of which is genetically distinct. Using this genetically diverse BXD panel, we mapped QTLs modulating GAS sepsis severity, and identified candidate genes within these QTLs that were parsed into pathways likely to modulate the severity of this complex infectious disease.

## Results

### Variable susceptibility to severe GAS sepsis in genetically distinct mice

Our initial studies [20] demonstrated that there is considerable variability among BXD strains with respect to susceptibility to severe GAS sepsis. To quantify differences among strains in this study, we used three main quantitative traits, namely animal survival, bacteremia, and bacterial dissemination to spleen.

**Figure 1:**
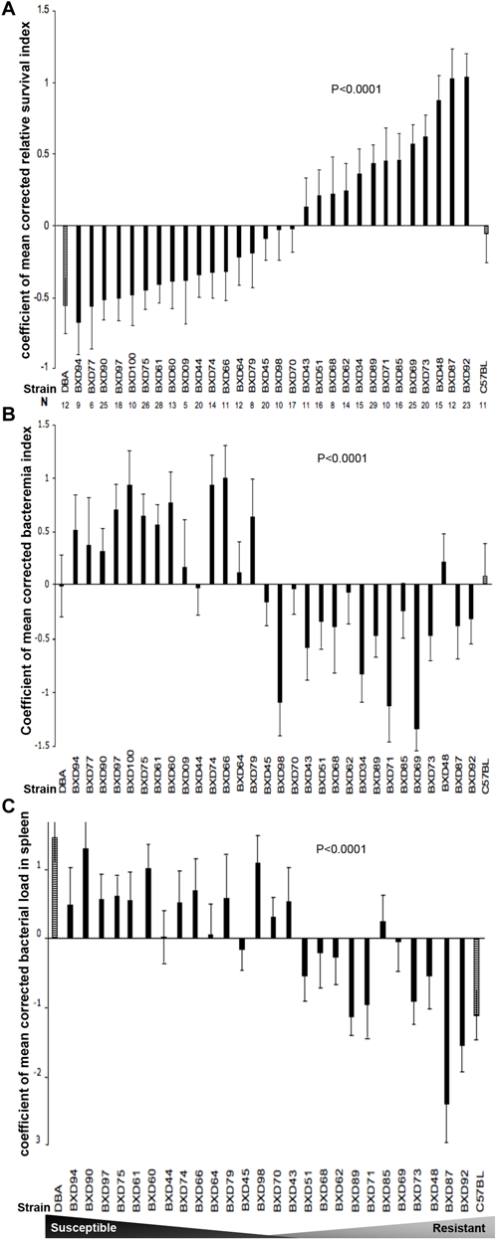
Differential susceptibility to GAS sepsis among different BXD strains and their parental strains. (A) Rank-ordered bar chart of survival expressed as mean values of coefficient of mean corrected relative survival indices (cRSI) across 30 different BXD strains and their parental strains (DBA/2J and C57Bl/6J) at the two extremes of the X-axis. Error bars represent the standard errors of the means. Statistical test is two-way ANOVA (with correction to covariates) P≤0.0001. Total number of mice is 489 belonging to 32 strains; number of mice used per strain is indicated. (B) Bar chart of GAS bacteremia expressed as coefficient of mean values of corrected log blood CFUs 24 h post injection (corrected log CFU/ml blood). Strains are ordered by their corrected relative survival indices with the parental strains at the two extremes of the X-axis. Error bars represent the standard errors of the means. Statistical test is two-way ANOVA (with correction to covariates) P≤0.0001. (C) Bar chart of GAS dissemination to spleen expressed as coefficient of corrected bacterial load in spleen (log CFU/spleen). Strains are ordered by their corrected relative survival indices with the parental strains at the two extremes of the X-axis. Error bars represent the standard errors of the means. Statistical test is two-way ANOVA (with correction to covariates) P≤0.0001.

We infected mice (n = 5–26 mice per strain), belonging to 32 strains (30 different BXD strains and their parental strains, B6 and D2), intravenously (i.v.) with the same bacterial dose (2±1.5×10^7^ CFU/mouse). Survival was expressed as normalized corrected relative survival indices (cRSI) calculated for each of the 32 strains as detailed in materials and methods. We observed significant difference in relative susceptibility to GAS sepsis across the BXD panel (P≤0.0001), Figure 1A. This wide range of susceptibility across the various strains, together with the finding that some of the strains showed phenotypes considerably more exaggerated than the parental strains (B6 and D2), is an illustration of how different combination of polymorphic genes can manifest quite differently according to the overall genetic context.

Similarly, we determined bacterial load in blood (log CFU/ml blood), 24 h post-infection and found considerable variation across the strains. In general there was an inverse correlation between mice survival and extent of bacteremia (*r* = −0.471, P≤0.0001, R^2^ = 22.2%), Figure 1B. Bacterial dissemination to spleen also varied across the strains, showing a stronger inverse correlation with mice survival (r = −0.717, P≤0.0001, R^2^ = 51.4%), Figure 1C.

Although these inverse correlations between mice survival and bacterial load in blood and spleen made biological sense because it is anticipated that susceptible strains would have higher bacterial load than in resistant strains, there were several exceptions. For example, although strains BXD44, and BXD45 are ranked susceptible based on their survival, they showed low bacterial load in blood and spleen. Similarly, BXD43 and BXD85 strains, which are ranked resistant, had a high bacterial load in their spleen. Another interesting observation was that, in general, susceptible strains survival was better correlated with bacterial load in blood and spleen than resistant strains. Together, these findings confirm that there is more than one mechanism modulating differential susceptibility or resistance to severe GAS sepsis [20].

### Genome-Wide Scans for Mapping GAS Susceptibility QTLs

We used bioinformatics tools available through Gene Network (GN) to link measured phenotypes to strains genotypes. Each of the quantified phenotypes was analyzed in the context of the studied mice genotypes and single nucleotide polymorphism (SNPs) using 3795 SNPs and microsatellite markers for BXD strains. Significant QTLs modulating survival, bacteremia 24 h post-infection, and bacterial dissemination to spleen mapped to mouse Chromosome (Chr) 2.

**Figure 2:**
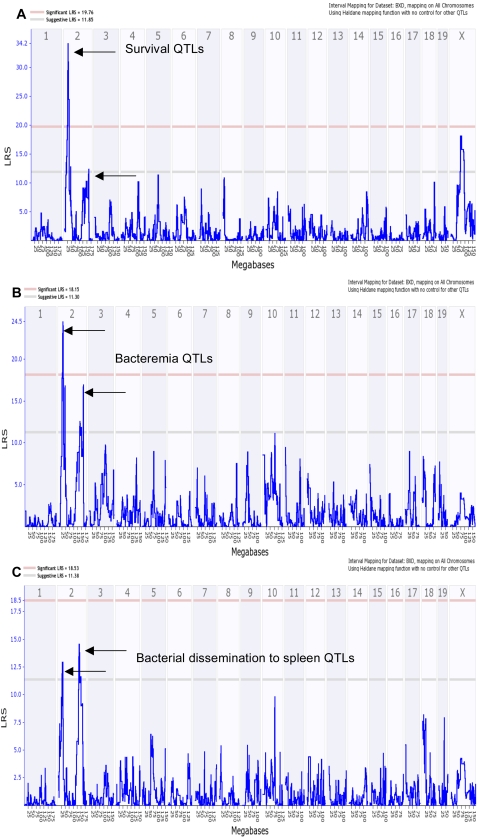
Genome-wide scan for mice susceptibility to GAS sepsis showing mapped QTL on Chr 2. (A) Interval mapping of survival (expressed as corrected relative survival index, cRSI), showing a significant QTL (based on 1000 permutation tests) on Chr 2 between 22–34 Mb with LRS of 34.2 (P≤0.0000001), and a suggestive QTL between 125–150 Mb with LRS of 12 (P≤0.001). (B) Whole genome interval mapping of GAS bacteremia using bacterial load 24 h post injection expressed as corrected log CFU/ml blood, showing two QTLs on Chr 2: first QTL between 22–34 Mb with LRS of 24.5 (P≤0.00001) and a second with LRS of 17 (P≤0.0001) between 125–150 Mb. (C) Whole genome mapping of GAS sepsis using tissue dissemination of GAS expressed as corrected log CFU/spleen with QTL between 125–150 Mb with LRS of 15 (P≤0.0001). Red line indicates significant LRS, while grey line indicates suggestive LRS. Upper x-axis shows mouse chromosomes, and lower x-axis shows physical map in mega bases, y-axis represents linkage in LRS score.

The strongest QTL modulating mouse survival (cRSI) mapped to mouse Chr 2 between 22–34 Mb, with an likelihood ratio statistic (LRS) of 34.2 (P≤0.0000001), Figure 2A. A second less significant QTL was also mapped on the same chromosome between 125–150 Mb with an LRS of 12 (P≤0.001), and a third QTL on Chr X, Figure 2A. The QTLs for bacteremia and bacterial dissemination to spleen overlapped with those for survival, with slight difference in significance. The first QTL modulating bacteremia mapped to Chr 2 between 22–34 Mb with an LRS of 24.5 (P≤0.00001), Figure 2B. The second QTL mapped to the same chromosome between 125–150 Mb with an LRS of 17 (P≤0.0001), Figure 2B. A QTL modulating bacterial dissemination to spleen also mapped to Chr 2 between 125–150 Mb with LRS of 15 (P≤0.001), Figure 2C.

To further investigate and narrow down the mapped region on Chr 2, we resorted to haplotype maps of BXD strains to select additional strains that may be informative in validating, and further confirming mapped QTLs.

### In silico prediction of BXD susceptibility to severe GAS sepsis

**Figure 3:**
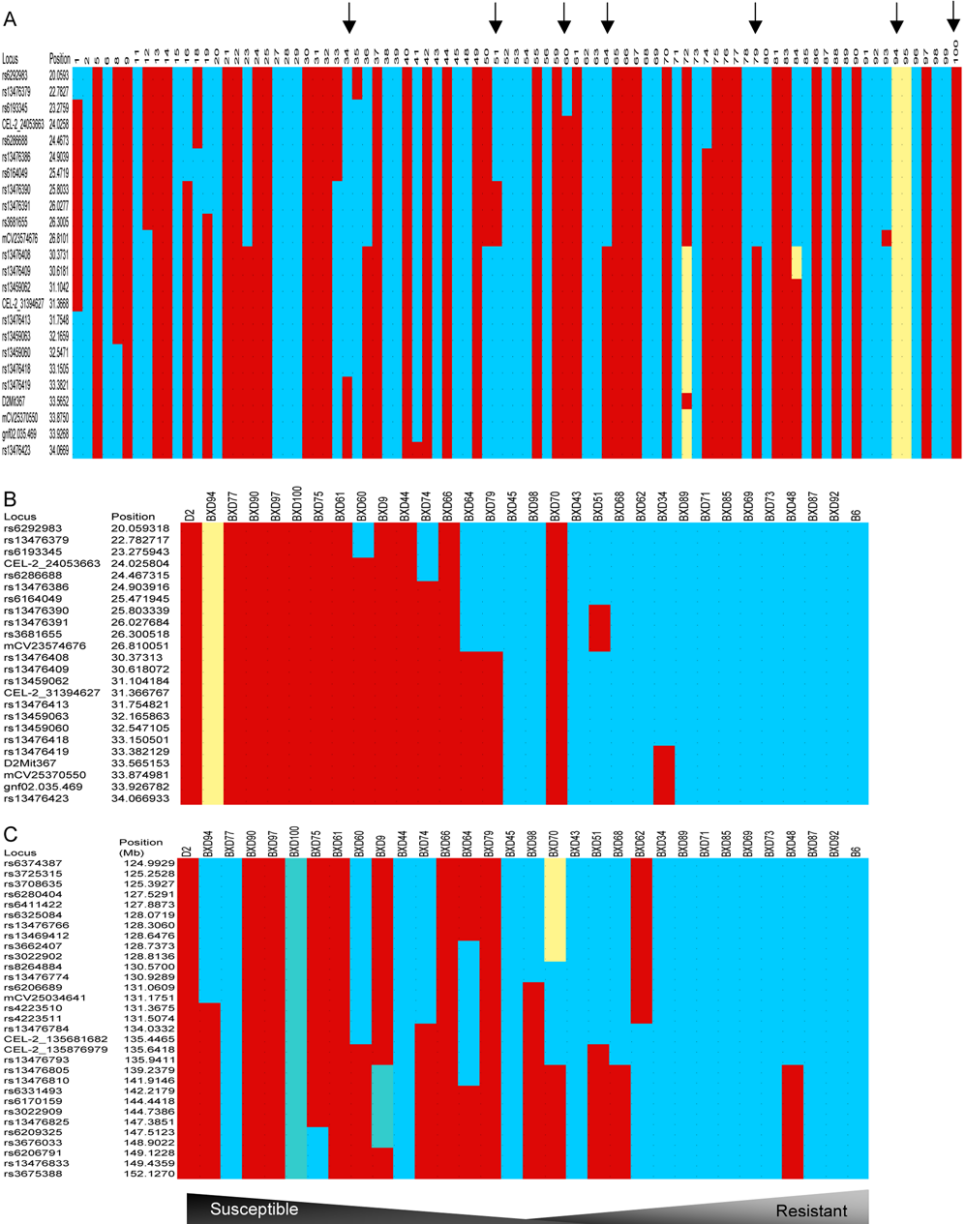
Recombinant inbred BXD strain distribution patterns at region of interest on Chr 2. (A) Haplotype maps of the first QTL on Chr 2 between 20–34 Mb showing available BXD strains. Haplotype maps were used for in silico selection of strains for survival studies based on the strain distribution patterns, where *B* allele (blue bars) inherited from the resistant parent C57Bl/6J and *D* alleles form the susceptible parent DBA/2J, beige bars indicate strains that are heterozygous at this region, resembling an F1. Arrows indicate BXD strains 34, 51, 60, 64, 79, 94, and 100. (B) Haplotype maps of the first QTL region on Chr 2 between 20—34 Mb across BXD strains used in the population-based survival experiments showing the pattern of alleles inherited from each parent at region of interest on Chr 2. The different BXD strains are rank-ordered according to their susceptibility to GAS sepsis form susceptible to more resistant. Resistant strains show a pattern of accumulation of alleles inherited from resistant parent (*B*) C57Bl/6J (blue bars) while the susceptible strains show a pattern of alleles from susceptible parent (*D*) DBA/2J (red bars), with the exception of BXD94 strain, heterozygous (beige bars) at the QTL region. (C) Haplotype maps of the second QTL region on Chr 2 between 125—150 Mb across BXD strains used in the population-based survival experiments showing the pattern of alleles inherited from each parent at region of interest on Chr 2. The different BXD strains are rank-ordered according to their susceptibility to GAS sepsis form susceptible to more resistant. BXD genotype data set can be downloaded from www.genenetwork.org/genotypes/BXD.geno.

Our mapped QTLs directed us to select more strains for survival experiments based on differences in their haplotypes within the QTLs. For example, we selected BXD100 with a *D* haplotype at Chr 2 between 22–34 Mb region, Figure 3A, and found it to be susceptible. Similarly, we tested BXD87 with a *B* haplotype in the same region and this strain as predicted, exhibited a resistant phenotype. To further narrow down the mapped QTLs, we chose strains with breakage in the mapped interval, i.e. with recombination at Chr 2 region 22–34 Mb for further survival testing (Figure 3A). BXD34 with a *B* haplotype at Chr 2 between 24–33.15 Mb region, was resistant, while BXD60 with a *B* haplotype at Chr 2 between 20–23.27 Mb region, was susceptible, Figure 1A and 3A. This narrowed down the relevant susceptibility region to Chr 2 between 23.27–33.15 Mb. Using more strains with recombination at this narrowed region, we found BXD51, 64, and 79 to be quite interesting as they showed intermediate resistance suggesting that genes at this region are candidate modulators of the mapped QTL, Figure 3B. Another interesting strain was BXD94 that we found susceptible to infection, yet has a heterozygous genotyping, suggesting that *D* allele exhibited dominance, Figure 3B.

Similarly, we inspected haplotypes of studied BXD strains at the second QTL between 125–150Mb (Figure 3C), the majority of susceptible strains had *D* alleles, while resistant strains had *B* alleles with some exceptions, for example BXD77 is a susceptible strain, yet had *B* alleles. It was interesting to find that the susceptible strain BXD44 had *B* alleles at this QTL (Figure 3C), suggesting that observed lower bacterial load in blood compared to other susceptible strains (Figure 1B) might be modulated by *B* allele in the second QTL. Meanwhile, BXD48, a resistant strain, has a breakage at 135.9 Mb (Figure 3C) with *D* alleles suggesting that its relatively higher bacterial load in blood (Figure 1B) might be modulated by the second QTL. Collectively, these findings suggest that both loci modulate different observed phenotypes of GAS sepsis severity.

### Mining for candidate genes and pathways in mapped loci

Based on our in silico strain selection and QTL validation by experimental assessment, we evaluated candidate genes within the mapped QTLs, taking into account linkage, gene ontology analyses, cocitation networks, and biological relevance. To categorize genes and transcripts in the mapped QTLs into functional pathways, we performed functional analyses using Ingenuity Pathways Analysis (IPA) (www.ingenuity.com). We parsed our genes into 50 functional networks; the most relevant networks involved those associated with immune response, cell signaling, cellular assembly and organization, and lipid metabolism. From these interrelated pathways, we selected 37 representative genes to study their role in GAS susceptibility QTL by quantitative analysis of their expression levels in selected resistant and susceptible strains (groups) pre- and post-infection (Table S1).

**Figure 4:**
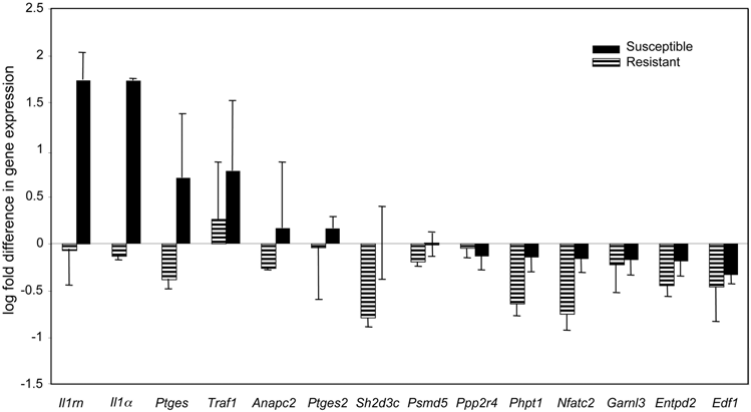
Patterns of differential gene expression levels of candidate genes post infection in susceptible and resistant strains. Quantitative PCR results showing levels of gene expression of 14 genes with significant (P = 0.05–0.08) change post-infection in susceptible and resistant strains, expressed as Log fold differences in gene expression level post-infection. Genes are grouped to three groups, a) genes up regulated in susceptible strains post-infection and decreased in resistant strains post-infection, b) genes down regulated in both susceptible and resistant strains post-infection, and c) genes up regulated in both susceptible and resistant strains post-infection. Susceptible strains are represented as solid black bars, and resistant strains as dashed bars. The bars represent 2–4 biological replicates run in three technical replicates each and significance is based on t-test. *Anapc2*, anaphase promoting complex subunit 2; *Entpd2*, ectonucleoside triphosphate diphosphohydrolase 2; *Edf1*, endothelial differentiation-related factor 1; *Garnl3*, GTPase activating RANGAP domain-like 3; *Il1a*, interleukin 1 alpha; *Il1rn*, interleukin 1receptor antagonist; *Nfatc2*, nuclear factor of activated t-cells, cytoplasmic, calcineurin-dependent 2; *Phpt1*, phosphohistidine phosphatase 1; *Ptges*, prostaglandin E synthase; *Ptges2*, prostaglandin E synthase 2; *Psmd5*, proteasome (prosome, macropain) 26S subunit, non-ATPase 5; *Ppp2r4*, Protein phosphatase 2A regulatory subunit B; *Sh2d3c*, SH2 domain containing 3C; *Traf1*, TNF a receptor associated factor 1.

We found that 28 out of a representative 37 genes were differentially expressed post-infection (Table S2), of those 14 genes were down regulated in resistant group while up regulated in susceptible group post-infection. Nine genes were down regulated in both groups post-infection, and five were up regulated in both groups post-infection (Table S2). Fourteen of the 28 differentially expressed genes showed significant change post-infection in both resistant and susceptible strains (Table 1 and Figure 4). In general, susceptible strains showed increase in the relative expression levels post-infection (19 genes) of both pro and anti-inflammatory genes, e.g. interleukin1 alpha (*Il1 α*), and Il1 receptor antagonist (*Il1rn*) respectively. By contrast, in the resistant BXD strains, most of the tested genes showed a decrease (23 genes) in expression levels post-infection with few exceptions (five genes) e.g. TNF receptor associated factor 1 (*Traf1*) (Figure 4 and Table S2). Differentially expressed genes were associated with both innate and early adaptive immune response. Among those associated with early immune response, *Il1a*, *Il1rn*, prostaglandin E synthase (*Ptges*), and *Ptges2* were up regulated in susceptible strains, whereas their levels decreased in resistant strains. Several of the differentially expressed genes show polymorphisms as SNPs between the parental strains B6 and D2, suggesting that these polymorphic genes modulate differential response to infection (Table S3). The differential expression of IL-1*α* was confirmed at the protein level (data not shown).

**Table 1:** The relative expression levels of candidate genes post infection in resistant and susceptible strains expressed as ratio of post/pre infection in susceptible and resistant strains.

Gene ID	Gene	Resistant strains	P value a	Susceptible strains	P value
Genes down regulated in resistant group while up regulated in susceptible group					
*Anapc2*	Anaphase promoting complex subunit 2	0.551	0.022*	1.475	0.509
*Il1 α*	Interleukin 1 α	0.739	0.010*	44.525	0.062 †
*Il1rn*	Interleukin 1receptor antagonist	0.851	0.680	54.687	0.221
*Ptges*	Prostaglandin E synthase	0.417	0.001 *	6.363	0.028 *
*Ptges2*	Prostaglandin E synthase 2	0.913	0.774	1.456	0.005 *
*Sh2d3c*	SH2 domain containing 3C	0.162	0.055 †	1.009	0.980
Genes down regulated in both resistant group and susceptible groups					
*Entpd2*	Ectonucleoside triphosphate diphosphohydrolase 2	0.359	0.010*	0.654	0.064 †
*Edf1*	Endothelial differentiation-related factor 1	0.349	0.095 †	0.470	0.014 *
*Garnl3*	GTPase activating RANGAP domain-like 3	0.601	0.142	0.674	0.070 †
*Nfatc2*	Nuclear factor of activated t-cells, cytoplasmic, calcineurin-dependent 2	0.178	0.095 †	0.693	0.208
*Phpt1*	Phosphohistidine phosphatase 1	0.229	0.077 †	0.714	0.233
*Psmd5*	Proteasome (prosome, macropain) 26S subunit, non-ATPase 5	0.644	0.006 *	0.963	0.652
*Ppp2r4*	Protein phosphatase 2A regulatory subunit B	0.902	0.256	0.734	0.091 †
Genes up regulated in both resistant group and susceptible group					
*Traf1*	TNF receptor associated factor 1	1.826	0.300	5.995	0.066 †

a Significance based on t-test,

*: P<0.05,

**†:** P = 0.06–0.08.

**Figure 5:**
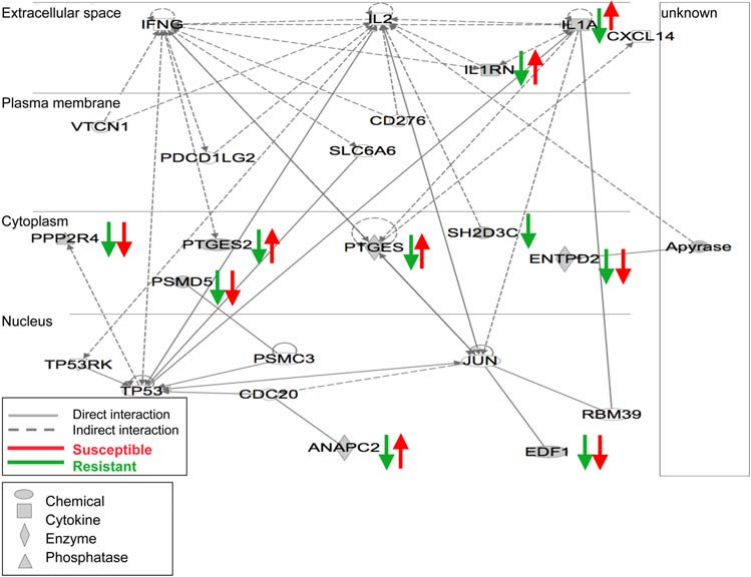
Functional network of genes modulating GAS QTL. Graphical representation of the molecular relationships between differentially expressed genes, showing the central role of IL-1, IL-1rn, PTGES, and PTGES2 in modulating response to GAS sepsis and their indirect interactions with IFN-γ and IL-2 networks in modulating bacterial sepsis. Genes are represented as nodes, and the biological relationship between two nodes is represented as line, solid lines represent direct interactions, dashed lines represent indirect interactions. Oval shapes represent chemical or drug, squares represent cytokines, diamond shapes represent enzymes, concentric circles represent group of family, and triangles represent phosphates. Blue lines and arrows represent expression levels of resistant strains, while red lines and arrows represent susceptible strains expression levels. Apyrase, ATP diphosphohydorlase; ANAPC2, anaphase promoting complex subunit 2; CDC20 cell division cycle homolog 20; CXCL14, chemokine (c-x-c motif) ligand 14; ENTPD2, ectonucleoside triphosphate diphosphohydrolase 2; EDF1, endothelial differentiation-related factor 1; JUN jun oncogene; IL1A, interleukin 1 alpha; IL1RN, interleukin 1receptor antagonist; IFNG interferon gamma; IL2 interleukin2; PDCD1LG2, programmed cell death ligand 2; PTGES, prostaglandin E synthase; PTGES2, prostaglandin E synthase 2; PSMC3 proteasome (prosome, macropain) 26S subunit ATPase 3; PSMD5, proteasome (prosome, macropain) 26S subunit, non-ATPase 5; PPP2R4, protein phosphatase 2A regulatory subunit B; RBM39, RNA binding protein 39; SH2D3C, SH2 domain containing 3C; SLC6A6, solute carrier family 6; TP53 tumor protein p53; TP53RK, TP53 regulating kinase; VTCN1, V-set domain containing T cell activation inhibitor 1.

We parsed the differentially expressed genes into pathways, using IPA, IL-1 and prostaglandins were key early response molecules modulating susceptibility to severe GAS sepsis in two the mapped networks, which are shown merged in Figure 5. The first network (P<10^−27^) comprised of genes related to lipid metabolism and innate immunity, e.g. *Il1a, Il1rn, Ptges*, while second network (P<0.01) contained genes modulating nucleic acid metabolism, energy production and host responses to injury e.g. Ectonucleoside triphosphate diphosphohydrolase 2 (*Entpd2*) (Figure 5).

## Discussion

It has been established that networks of multiple pathways, rather than a single gene, modulate traits and affect susceptibility and outcomes of virtually all diseases. The ARI strains used in this study afford one of the best unbiased forward genetics approaches to determine how different combinations of polymorphic genes interact to shape disease phenotypes. Using this panel of genetically diverse reference population, we were able to map QTLs modulating specific phenotypes associated with severe GAS sepsis.

GAS causes a wide range of diseases depending on multiple factors including site and route of infection, interplay of the pathogen virulence factors with host immune defenses that are affected by the host immune status and difference in the genetic make up of the host [6]. Thus, these bacteria represent a good model to explore the impact of host complex traits on susceptibility to infections. Previous studies have shown that host-pathogen interactions modulate the severity of GAS sepsis [5],[6],[9],[10], and we found that patients with GAS sepsis expressing HLA class II DRB1*15/DQB1*06 (DR15/DQ6) haplotype are protected from severe GAS sepsis, whereas those with HLA class II DRB1*14/DQB1*05 (DR14/DQ5) haplotype are at high risk for developing severe and often fatal forms of the disease [6]. The strong association between disease severity and HLA class II allelic polymorphism is primarily related to the differential ability of HLA class II alleles to present SAgs to T cells, where presentation of Strep SAgs by the protective HLA class II alleles results in a significantly attenuated response as compared to their presentation by the neutral or high-risk alleles [6],[8],[21]. While this association made perfect biological sense based on the known central role of SAgs in the human disease, mice are not susceptible to SAgs due to an inherent lower affinity of mouse MHC class II molecules to GAS SAgs. The role of GAS SAgs can be well investigated in HLA class II transgenic mice as others and we have reported [8],[12],[22],[23]. However, due to the overwhelming response to SAgs in these mice, it is difficult to use them to dissect host response to other GAS virulence factors in the disease process. For this reason, our present ARI mouse model of sepsis is ideal for identifying host genetic variations, besides the MHC class II antigens, that may also contribute to differences in GAS sepsis severity.

We invested time and effort to standardize the GAS infection model using a large number of BXD mice (n = 717) and this allowed us to optimize infection dose and identify significant covariates (e.g. age and sex) needed to be accounted for in our final analysis [20]. Although GAS strains can vary considerably with regards to virulence, we showed that the same virulent strain could cause diseases with starkly different severity in humans [5],[6]. The strain used in this study is a hypervirulent derivative of the M1T1 clonal strain that emerged in the 1980's at the same time that the severe forms of the invasive GAS infections resurged [3], [24]–[26]. Initial studies [20] identified an optimal infection dose of 2±1×10^7^ CFU/mouse, and indicated the need to use mice with an age range of 40–120 days for linear correlation with survival. In addition, these pilot studies determined that sex has insignificant effect in this GAS sepsis model [20] and revealed a stark variation among various BXD strains used with respect to their susceptibility to severe sepsis. However, precise mapping of QTLs required that we study more BXD strains and include more mice per BXD strain to obtain statistical power.

With a total of 30 BXD strains and an average of 15 mice per strain, we mapped QTLs on Chr 2 that modulate severity to GAS sepsis, measured by comparing mice survival post-infection, bacterial load in blood, and bacterial dissemination to spleen across the BXD strains. Inasmuch as the BXD strains are heavily genotyped with more than 3600 genomic markers, identifying QTLs is relatively straightforward. The mapped QTLs on Chr 2 harbor a relatively large number of candidate genes associated with various functional networks and signaling pathways including nuclear factor-κB (NF-κB) and p38 mitogen activated protein kinases (MAPK) pathways, proliferation of immune cells, and eicosanoid signaling. Such output is typical of unbiased genome-wide analysis studies, and required further analyses to determine which of these genes are the key modulators of the studied trait.

To narrow down the gene list to a handful of genes that can be experimentally validated, we used multiple methods including linkage, gene ontology, and differential gene expression analyses. Our quantitative PCR analysis of 37 representative candidate genes showed that 28 genes were differentially expressed in selected susceptible and resistant strains post-infection. These differentially expressed genes fell into three main categories, genes associated with innate and adaptive immune response, and genes associated with apoptosis. Differentially expressed genes associated with innate immune response were both pro- and anti-inflammatory as well as adaptive immune response genes associated with T and B cell proliferation and differentiation, cell signaling and antigen processing and presentation.

Differentially expressed genes associated with innate immune response were either related to pro- or anti-inflammatory responses. Both pro- and anti-inflammatory associated genes were up-regulated in the selected susceptible strains post-infection, and this is in agreement with a recent study by Goldmann, et al. [27] who showed a mixed pro- and anti-inflammatory response belonging to M1 and M2 macrophage phenotypes in response to GAS infection. This increase in both pro- and anti-inflammatory response could be attributed to homeostatic mechanisms where, for example, the increase in *Il1* levels in the susceptible strains was accompanied by an increase in its endogenous antagonist *Il1rn*. This was not the case in the resistant strains that showed decreased levels of expression of pro- and anti-inflammatory related genes both pre- and post-infection. These findings suggest that susceptibly to GAS sepsis is associated with an overzealous innate immune response as observed in susceptible BXD strains only. These results mirror previous findings in humans, where association of exaggerated inflammatory responses, including IL-1, with susceptibility to GAS sepsis was detected [6],[28],[29]. However, unlike what we found in this mouse model, human responses are dominated by high levels of IFN-γ and TNF-ß, presumably because the human disease is driven primarily by SAgs [29],[30].

In general, differentially expressed genes associated with early adaptive immune responses showed a pattern of decrease in expression levels in both susceptible and resistant strains with the exception of anaphase promoting complex subunit 2 (*Anapc2*) that was slightly increased in susceptible strains post-infection (Figure 5). By contrast, several genes associated with pro- and anti-apoptotic response were differentially expressed in the selected susceptible and resistant strains post-infection (Figure 5). Apoptosis in streptococcal pathogenesis is affected by interacting factors including, context of infection [31], cells undergoing apoptosis [32]–[34], for example, apoptosis aids in the clearing of infection if macrophages are undergoing apoptosis, while it would be harmful to the host if lymphocytes are undergoing apoptosis. Other factors include, whether the bacteria is internalized or extracellular [35] and accordingly the type of apoptosis pathways activated [36]. In our murine GAS sepsis model, we have measured expression levels in whole spleens, which involved the response of various cells including macrophages, T and B lymphocytes, dendritic and endothelial cells etc. Consequently, we expected to observe a mixed response; however, in our ongoing studies, we are examining possible alterations in splenic population profiles post-infection in the various BXD strains to determine which cell types are responsible for the major differences in cytokine levels seen post-infection and to dissect the role of different cell populations in this GAS sepsis model.

Another interesting observation was that the relative expression level of genes measured in the selected resistant strains showed a pattern of decrease post-infection, with the exception of five genes, heat shock 70KDa protein 5 (*Hspa5*), *Traf1, Traf2*, Notch gene homolog 1 (*Notch1*), and signal-regulatory protein alpha (*Sirpa*). It is worthy to note the link between these genes is as they are involved directly and indirectly with activation of early adaptive response. *Hspa5* is an Hsp70, which is associated with cytoprotection, anti-apoptosis, and anti-inflammation [37]–[39], and has been associated with immune response to sepsis [40],[41]. *Notch1* has been associated with the signaling involved in regulation of lymphocytes development and activation to effector cells [42], natural killer cells development [43]–[45] and recently *Notch1* was associated with macrophage activation [46].We took into consideration in our experimental design that stress might alter the expression of stress related genes especially heat shock proteins and chemokines, therefore, we subjected control mice to the same stress as infected mice by injecting control mice with saline, so that any change in expression levels would be accounted to GAS sepsis.

Among the differentially expressed genes, four genes, *Il1, Il1rn, Ptges,* and *Ptges2*, showed marked up regulation in susceptible strains, while showed no change or slight decrease in levels in resistant strains post-infection (Figure 5). Expression of *Ptges* and *Ptges2* genes increase is induced by the increased levels of *Il1* [47], which is an indirect effect of the activation of IFN-γ (Figure 5). Ptges and Ptges2 are prostaglandins synthases for the lipid inflammatory mediators PGE2, which along with platelet activating factor and leukotriens, mediate vasodilatation in the early response to inflammation [48],[49]. Vasodilatation in turn leads to hypotension, a hallmark of STSS. Although the role of prostaglandins in inflammation and immune response has long been studied, their role in the immune response to infectious diseases has been lately pursued [50]–[55]. Increased production of prostaglandins has been associated with various Gram positive bacterial infections including *Streptococcus suis* [53], group B streptococcal [55], and GAS skin infections [54].

In conclusion, our holistic approach of studying the genetic basis of differential susceptibility to GAS sepsis revealed loci on Chr 2 that harbor major immune modulators. In the present study, we examined the interactions of pathogen multiple virulence factors with the host immune system in an *in vivo* model of sepsis using a genetically diverse reference population. This shed light on a network of host genes modulating variation in GAS severity, which includes cytokines, pro- and anti-inflammatory mediators, and genes associated with apoptosis and early adaptive immune response. Our ongoing detailed studies to identify interactive molecular pathways contributing to the complex trait of GAS sepsis will undoubtedly help us dissect the various mechanisms by which the host interacts with the bacteria *in vivo*, resulting in resistance or increased susceptibility to severe GAS sepsis.

## Materials and Methods

### Mice

We generated the BXD advanced recombinant inbred (ARI) mice at UTHSC by crossing B6 and D2 mice to generate F1 hybrids, which were crossed to generate F2 progeny, each with random patterns of recombination. Random crossing of F2 mice generated F3 and so on, till F11 generation, after which we designated pairs of F11 hybrids as parents of each BXD line, and inbred them by sib mating for >20 generations to achieve homozygosity for each genetically distinct BXD line. This breeding scheme was done to increase recombination events resulting in roughly double the number of recombinations per strain compared to conventional RI strains [56],[57].

The genomes of the B6 and D2 parental strains have been sequenced and a database of their SNPs is available at the Gene Network (GN) web site (www.genenetwork.org/webqtl/snpBrowser.py). Simple sequence length polymorphism (SSLP) markers were typed for all BXD RI strains as previously described [56]. The BXD progeny is genotyped at 13377 SNPs and microsatellite markers [58], a selected subset of 3795 SNPs and microsatellite markers used by GN BXD genotype dataset for mapping traits, can be downloaded at www.genenetwork.org/genotypes/BXD.geno.

We recently standardized the model of BXD ARI mice for use in GAS infection studies [20]. In the current study, a total of 696 mice were used, from which 183 flagged mice were excluded based on predetermined criteria as previously detailed in [20].

All procedures involving mouse tissues were approved by the institutional animal care and use committee at the UTHSC.

### Experimental design

#### A) Population-based experiments

Groups of 5–26 mice from a total of 30 BXD lines and their parental strains (B6 and D2) were injected via tail vein with 2±1.5×10^7^ CFU/mouse of a hypervirulent form of the clinical isolate GAS 5448 (M1T1) [5],[24],[59] as detailed previously [20]. Mortality and weight loss were recorded every 8 h for the ensuing 6 days. To normalize across experiments, we inspected survival days distribution clusters for each experiment and determined multimodal distribution and boundaries of each cluster for a total of three clusters: susceptible, intermediate, and resistant. Survival days within each cluster were then converted into a survival index ranging from 0.25–1, 1.25–2, and 2.25–3 for susceptible, intermediate, and resistant clusters respectively. The survival index was assigned to each mouse irrespective of its strain. Indices for each strain, across experiments were then corrected for significant covariates (age, sex, body weight, and inocula) using multiple regression analyses.

#### B) Phenotype assessment experiments

We designed experiments for the assessment of survival, bacteremia, and bacterial dissemination to spleen. We assessed relative mice survival and bacteremia in the same experiment. We measured bacteremia as CFU/ml by culturing serial dilutions of blood drawn from mice tails 24 h post-injection. We determined bacterial load in spleen by homogenizing spleens of expired mice using tissue homogenizer, TH (Omni International, Marietta, GA). Each tissue homogenate was serially diluted 10 folds to 10^−6^ fold, and each dilution was cultured for determination of bacterial load in spleens (CFU/ml) that was then normalized to spleen weight and expressed as CFU/spleen.

### QTL Mapping

We performed QTL mapping using web-based complex trait analysis available on the GN website and the mapping module which analyzes phenotypes in context of mouse genotypic differences. Interval mapping evaluates potential QTL at regular intervals and estimates the significance at each location using 1000 permutation tests [60]. We performed three sets of analyses using strain means for the following three variables: (1) corrected relative survival index, described above, (2) log bacterial load in blood 24 h post injection, and (3) log bacterial load in spleen at expiration.

### Quantitative PCR analysis for target genes expression

We investigated the differential expression of target genes in spleen of infected vs. control PBS-injected mice at selected time points, we selected strains based on their susceptibility, BXD61 and 90 representing susceptible strains, and BXD73 and 87, representing resistant strains. We performed 2–3 biological replicates of each set of paired susceptible and resistant (n = 6–8 per strain). Mice were sacrificed 40 h post i.v. injection with 2±1.5×10^7^ CFU/mouse of clinical isolate GAS 5448 (M1T1) and RNA from individual mice was extracted from spleens. Bacteremia was determined as described above. We isolated RNA using RNA-STAT 60 method and when necessary, we purified RNA samples using RNeasy mini kit clean up columns (Qiagen, Valencia, CA). We pooled RNA samples per strain with A260/280 ratios≥1.8 for cDNA synthesis with SuperScript III reverse transcriptase kit (Invitrogen, Carlsbad CA) using oligo dT primers. We designed real time PCR assays, hydrolysis probes, and gene specific primers that span long introns to distinguish cDNA from genomic DNA using primer design online software universal probe library (UPL) at www.roche-applied-science.com/sis/rtpcr/upl/index.jsp. We performed quantitative TaqMan PCR on light cycler LC480 (Roche Applied Science, Indianapolis, IN). We used the mouse housekeeping gene, hypoxanthine guanine phosphoribosyl transferase (*Hprt1*) as an endogenous control to which we normalized gene expression data. Primer sequences are listed in Table S1. Samples were analyzed in triplicates for each of 2–4 biological replicates. We used delta delta Ct (threshold cycle) method for calculating relative expression levels expressed as fold differences between pre- and post-infection values for each gene analyzed. Student t-test was used to assess statistical significance.

### Identification of differentially expressed genes in the mapped interval and bioinformatics functional pathways analyses

We generated functional analyses of genes within the QTLs using Ingenuity pathways analysis (IPA) (www.ingenuity.com). Each data set containing gene identifiers was uploaded into the online application, and each gene was overlaid onto a molecular network developed from information contained in the ingenuity pathways database. Networks of genes were then generated based on their connectivity, and we chose the top 50 significant networks. The significance of the association between the data set and the pathways was measured in two ways: (1) the ratio of the number of genes from the data set that map to the pathway divided by the total number of genes that map to the pathway; and (2) by Fischer's exact test with P<0.001.

#### Statistical analysis

We used the DataDesk 6.2 (www.datadesk.com) to calculate mean survival indices, corrected survival indices, and correlation coefficients. Covariates association was evaluated for significant associations by Spearman correlation as described previously [20]. Fischer exact test was used to analyze association of the analyzed differentially expressed genes with specific pathways.

### Web site URL

Our data sets stored in WebQTL can be found at Gene Network (www.genenetwork.org) under BXD published phenotypes record ID 10836.

## Supporting Information

Table S1 Primer sequences used in quantitative PCR assays for candidate genes.
(0.08 MB DOC)

Table S2 Relative expression levels of candidate gene list expressed as mean fold difference between pre- and post-infection±standard deviation (SD) in selected resistant and susceptible strains.
(0.09 MB DOC)

Table S3 SNP analysis between parental strains C57Bl/6J and DBA/2J of selected genes in the mapped QTLs.
(0.05 MB XLS)
