# Supplementary material for: An Unbiased Systems Genetics Approach to Mapping Genetic Loci Modulating Susceptibility to Severe Streptococcal Sepsis
Source: PLoS Pathog. 2008 Apr 18;4(4):e1000042. doi: 10.1371/journal.ppat.1000042 (PMC2277464; doi:10.1371/journal.ppat.1000042)
Supplement: Table S1 — Primer sequences used in quantitative PCR assays for candidate genes. (0.08 MB DOC) [file ppat.1000042.s001.doc]

**Table S1.** Primer sequences used in quantitative PCR assays for candidate genes.

| **Gene** | **Gene description** | **Accession number** | **Forward 5’** | **Reverse 3’** |
| --- | --- | --- | --- | --- |
| *Anapc2‭‬* | Anaphase promoting complex subunit 2 | NM_175300 | ctcaaggtggccctagagact | agagtgatgatgtcacatgtgttg |
| *Asb6‭‬* | Ankyrin repeat and SOCS box-containing protein 6 | NM_133346 | aggagcccctggatgatt | cagctccgtgaggaccag |
| *Edf1‭‬* | Endothelial differentiation-related factor 1 | NM_021519 | ccgcacaggccaagtcta | aatggaatgctgcttgttctg |
| *Entpd2‭* | Ectonucleoside triphosphate diphosphohydrolase 2 | NM_009849 | cagagaccacctgcaacca | ggtctgctgtccgggtact |
| *Fbxw2‭‬* | F-box and WD-40‭ ‬domain protein 2 | NM_013890 | caaagtcaagtctctcttgcaca | tcccaattggccaaatctt |
| *Garnl3* | GTPase activating RANGAP domain-like 3 | NM_178888 | tccaccatgttgccatattc | caatgtggcgcttccttt |
| *Gpr107‭‬* | G protein-coupled receptor 107 | NM_178760 | cctgcagctctctcaggaag | cacacccgatgtcgtcacta |
| *Hspa5‭‬* | Heat shock 70kD protein 5‭ (‬glucose-regulated protein‭)‬ | NM_022310 | ctgaggcgtatttgggaaag | cagcatctttggttgcttgtc |
| *Il1 α* | Interleukin 1 α | NM_010554.4 | ttggttaaatgacctgcaaca | gagcgctcacgaacagttg |
| *Il1rn* | Interleukin 1 receptor anatagonist | NM_031167 | tgtgccaagtctggagatga | ttctttgttcttgctcagatcagt |
| *Mapkap1‭‬* | Mitogen-activated protein kinase associated protein 1 | NM_177345 | accttgccgaggagaagag | gcgtcggactcgaagtacag |
| *Nfatc2* | Nfatc2 nuclear factor of activated t-cells, cytoplasmic, calcineurin-dependent 2* | NM_010899  NM_001037177  NM_001037178 | caggaggacaccctgtgg | tcatctgctgtcccaatgaa |
| *Notch1‭‬* | Notch gene homolog 1‭ (‬Drosophila‭)‬ | NM_008714 | tgcaactgtcctctgccata | gtagcacatggggccaac |
| *Noxa1‭‬* | NADPH oxidase activator 1 | NM_172204 | atctggagcccatggattt | gcgttgtggtcatcaggaat |
| *Phpt1‭‬* | Phosphohistidine phosphatase 1 | NM_029293 | cagagccaggacaggaagata | ttggctttgatcttctcagttg |
| *Phyhd1* | Phytanoyl-CoA dioxygenase domain containing 1 | NM_172267 | gtctggagcccagggaat | gggcctttagctgtagagtgg |
| *Ppp2r4* | ‭Protein phosphatase 2A‭, ‬regulatory subunit B‭ | NM_138748 | ggcagctcacagctcataga | gccttctcatccacgaaatg |
| *Psmd5‭‬* | ‭‬Proteasome‭ (‬prosome‭, ‬macropain‭) ‬26S subunit‭, ‬non-ATPase‭, ‬5‭ | NM_080554 | gacgggtgaggacgtgtta | cgtccgtgatgggtataagc |
| *Ptges* | Prostaglandin E synthase | NM_022415 | gcacactgctggtcatcaag | acgtttcagcgcatcctc |
| *Ptges2‭‬* | Prostaglandin E synthase 2 | NM_133783 | cccaggaaggagacagctt | aggtaggtcttgagggcactaat |
| *Rab14‭‬* | ‭RAB14‭, ‬member RAS oncogene family‭ | NM_026697 | aggtgcgctcatggtgtat | caaccagctgcttaagtggtta |
| *Sh2d3c* | SH2‭ ‬domain containing 3C | NM_013781 | cagatcccagatctccactca | agcatggactcgggttacag |
| *Sirpa* | Signal-regulatory protein α | NM_007547.2 | agggagcatgcaaaccttc | tttgatccggaggaggtaga |
| *Traf1* | TNF receptor associated factor 1 | NM_009421 | gagcacatcctgagcttgg | tctttttgagccagggtttg |
| *Traf2* | TNF receptor associated factor 2 | NM_009422 | gctccttctgcctgacca | agacacaggcagcacagttc |
| *Tubb2c1* | ‬Tubulin‭ β 2c‭ | NM_146116.1 | gctcctcttctacagctgttcc | cgctgattacctcccagaact |
| *Ubadc1‭‬* | Ubiquitin associated domain containing 1 | NM_133835 | gaacaacaaccagcagaacg | tccaattcctcaggggatg |
| *Urm1‭‬* | Ubiquitin related modifier 1‭ ‬homolog‭ (‬S‭. ‬cerevisiae‭)‬ | NM_026615 | gagcgaccagagctgttcat | ggcatcattaatcagcacca |

* Nfatc2 has three transcript variants; we designed a common assay for the three transcripts, and differentiating assays for each isofrom. All assays showed similar results, shown are primers sets of common assay for the three transcript variants.
