# Supplementary material for: An Unbiased Systems Genetics Approach to Mapping Genetic Loci Modulating Susceptibility to Severe Streptococcal Sepsis
Source: PLoS Pathog. 2008 Apr 18;4(4):e1000042. doi: 10.1371/journal.ppat.1000042 (PMC2277464; doi:10.1371/journal.ppat.1000042)
Supplement: Table S2 — Relative expression levels of candidate gene list expressed as mean fold difference between pre- and post-infection±standard deviation (SD) in selected resistant and susceptible strains. (0.09 MB DOC) [file ppat.1000042.s002.doc]

**Table S2.** Relative expression levels of candidate gene list expressed as mean fold difference between pre- and post-infection ± standard deviation (SD) in selected resistant and susceptible strains.

| Gene ID | Gene | Resistant strains | SD | Susceptible strains | SD | |
| --- | --- | --- | --- | --- | --- | --- |
| Genes down regulated in resistant group while up regulated in susceptible group | | | | | |  |
| *Anapc2* | Anaphase promoting complex subunit 2 | 0.551 | 0.022 | 1.475 | 0.691 | |
| *Asb6* | Ankyrin repeat and SOCS box-containing protein 6 | 0.573 | 0.482 | 2.300 | 1.801 | |
| *Fbxw2* | F-box and WD-40 domain protein 2 | 0.930 | 0.384 | 1.159 | 0.268 | |
| *Gpr107* | G protein-coupled receptor 107 | 0.866 | 0.481 | 2.196 | 0.758 | |
| *Il1 α* | Interleukin 1 α | 0.739 | 0.045 | 54.687 | 0.011 | |
| *Il1rn* | Interleukin 1 receptor anatagonist | 0.851 | 0.384 | 55.909 | 0.274 | |
| *Mapkap1* | Mitogen-activated protein kinase associated protein 1 | 0.732 | 0.199 | 1.189 | 0.460 | |
| *Noxa1* | NADPH oxidase activator 1 | 0.270 | 0.164 | 1.086 | 0.151 | |
| *Phpt1* | Phosphohistidine phosphatase 1 | 0.229 | 0.133 | 0.714 | 0.155 | |
| *Ptges* | Prostaglandin E synthase | 0.417 | 0.101 | 5.046 | 0.679 | |
| *Ptges2* | Prostaglandin E synthase 2 | 0.913 | 0.553 | 1.456 | 0.122 | |
| *Rab14* | RAB14, member RAS oncogene family | 0.704 | 0.268 | 1.411 | 0.711 | |
| *Sh2d3c* | SH2 domain containing 3C | 0.162 | 0.102 | 1.009 | 0.389 | |
| *Phyhd1* | Phytanoyl-CoA dioxygenase domain containing 1 | 0.806 | 0.727 | 1.288 | 0.288 | |
| *Urm1* | Ubiquitin related modifier 1 homolog (S. cerevisiae) | 0.834 | 0.625 | 1.277 | 0.309 | |
| Genes down regulated in both resistant group and susceptible groups | | | | | |  |
| *Entpd2* | Ectonucleoside triphosphate diphosphohydrolase 2 | 0.359 | 0.114 | 0.654 | 0.160 | |
| *Edf1* | Endothelial differentiation-related factor 1 | 0.350 | 0.375 | 0.470 | 0.109 | |
| *Garnl3* | GTPase activating RANGAP domain-like 3 | 0.601 | 0.292 | 0.674 | 0.158 | |
| *Nfatc2* | Nuclear factor of activated t-cells, cytoplasmic, calcineurin-dependent 2 | 0.178 | 0.174 | 0.693 | 0.147 | |
| *Psmd5* | Proteasome (prosome, macropain) 26S subunit, non-ATPase, 5 | 0.644 | 0.049 | 0.963 | 0.123 | |
| *Ppp2r4* | Protein phosphatase 2A, regulatory subunit B | 0.902 | 0.108 | 0.734 | 0.150 | |
| *Ubac1* | Ubiquitin associated domain containing 1 | 0.865 | 0.960 | 0.730 | 0.971 | |
| *Tubb2c* | Tubulin β 2c | 0.846 | 0.506 | 0.925 | 0.477 | |
| Genes up regulated in both resistant group and susceptible group | | | | | |  |
| *Hspa5* | Heat shock 70kD protein 5 (glucose-regulated protein) | 1.717 | 1.016 | 4.040 | 2.587 | |
| *Notch1* | Notch gene homolog 1 (Drosophila) | 2.005 | 1.192 | 10.424 | 7.833 | |
| *Traf1* | Tnf receptor-associated factor 1 | 1.826 | 0.596 | 5.995 | 0.733 | |
| *Traf2* | Tnf receptor-associated factor 2 | 2.369 | 2.694 | 2.421 | 0.844 | |
| *Sirpa* | Signal-regulatory protein α | 4.092 | 6.024 | 1.607 | 1.034 | |
